# Do the therapeutic vaccines hold hope for the treatment of hepatitis B?

**DOI:** 10.1007/s12072-025-10907-2

**Published:** 2025-09-27

**Authors:** Qiujing Yan, Xinghuan Fu, Yan Wang, Guiqiang Wang

**Affiliations:** 1 Department of Infectious Disease, Center for Liver Disease, Peking University First Hospital, Beijing, China; 2 Department of Infectious Diseases, Peking University International Hospital, Beijing, China

**Keywords:** Chronic hepatitis B, Therapeutic vaccines, Immune tolerance, Functional cure

## Abstract

**Background:**

Chronic hepatitis B (CHB) is a global disease that can induce severe conditions such as cirrhosis and liver cancer, posing a serious threat to human health. The low functional cure rate of existing antiviral treatment drugs is primarily due to the dysfunctional immune response in CHB patients and the difficulty in clearing intrahepatic cccDNA.

**Methods:**

This article reviews the latest progress in the research on therapeutic vaccines for CHB, to analyze and discuss the potential impact of current achievements and to outline the prospects for future research, exploring therapeutic strategies for the functional cure of CHB.

**Results:**

Therapeutic vaccines are anticipated to activate specific immune responses in CHB patients, break the mechanisms of immune tolerance, and exhibit significant potential in maintaining long-term immune activity. Several therapeutic vaccines have already demonstrated promising therapeutic effects.

**Conclusions:**

Therapeutic vaccines are of great significance in the field of CHB treatment. Current research achievements provide strong support for their application, while combined therapy offers new ideas and hope for future treatment directions.

**Supplementary Information:**

The online version contains supplementary material available at 10.1007/s12072-025-10907-2.

## Introduction

In 2024, the World Health Organization (WHO) statistics reported that 254 million people worldwide are infected with hepatitis B virus (HBV), with approximately 1.2 million new infections, and about 80% cirrhosis and hepatocellular carcinoma (HCC) induced by chronic hepatitis B (CHB) which is a serious threat to human health [1]. After HBV infection, covalently closed circular DNA (cccDNA) is formed in hepatocytes’ core, and the viral genes may integrate into the host genome. These processes contribute to the persistence of HBV and make a significant challenge for the development of effective antiviral therapies. Recently, a meta-analysis conducted by the APASL Viral Elimination Taskforce, which included 1789 non-cirrhotic HBV patients, revealed that novel antiviral therapies achieved a low rate of HBsAg loss with 0.9% at the end of treatment and 0.1% at the end of follow-up. Notably, there was a significant rebound in HBsAg levels in 20.5% of cases off-treatment [2]. So that there is still no drug that can completely cure hepatitis B. What’s more, the Janssen Research & Development LLC, GlaxoSmithKline plc and Roche pharmaceutical companies have terminated HBV related drug development in 2023–2024 (Table 1), including HBV siRNA, PD-1/PD-L1, TLR agonist, core assembly modulator, HepTcell, and HBsAg monoclonal antibody. The main reason might be  that these drugs failed to achieve the desired goal of functional cure for CHB, especially the HBsAg clearance.

**Table 1:** Summary of recently terminated HBV-related treatment projects

NO.	Drug code	Drug type	Clinic trail	Phase	Manufacturer	Terminal time
1	RG6449（RO7565020）	Anti-HBsAg, IgG1 monoclonal antibody	NCT05763576	I	Roche	2024.12
2	RG7854（RO7020531）	TLR agonist	NCT04225715	II	Roche	2024.12
3	RG6346（RO7445482）	siRNA	NCT04225715	II	Roche	2024.12
4	RG6084（RO7191863)）	PD-L1/PD-1 targeted single strand oligonucleotide	NCT04225715	II	Roche	2024.12
5	HepTcell	polypeptide	NCT04684914	II	Altimmune	2024.6
6	GSK3528869A	viral vector and adjuvant protein	NCT03866187	I/II	GlaxoSmithKline plc	2024.12
7	JNJ-64457744	Small molecule chemical drugs	NCT05423106	I	Janssen Research & Development LLC	2023.7
8	JNJ-63723283	PD-1 monoclonal antibody	NCT05818683	I	Janssen Research & Development LLC	2023.7
9	JNJ-64530440	Capsid assembly regulator	NCT03915886	I	Janssen Research & Development LLC	2023.7
10	JNJ-64794964	TLR7 agonist	N/A	I	Janssen Research & Development LLC	2023.7
11	JNJ-56136379	Capsid assembly regulator	NCT04853524	IIb	Janssen Research & Development LLC	2022

Given the significant challenges and difficulties, is there still hope for the treatment of hepatitis B? The molecular mechanisms for immune clearance of HBV cccDNA and integrated DNA remain unclear till now, but the favorable prognosis observed in acute hepatitis B patients provides the valuable evidence to develop therapeutic HBV vaccines. The therapeutic vaccines could activate the immune response, induce HBVspecific immunity and help to break immune tolerance. Based on individualized treatment, exploring combination therapy regimens grounded in the diverse mechanisms of therapeutic vaccines and drugs may hold the hope for the HBV “Functional treatment” and is expected to become a new trend.

**Fig. 1:**
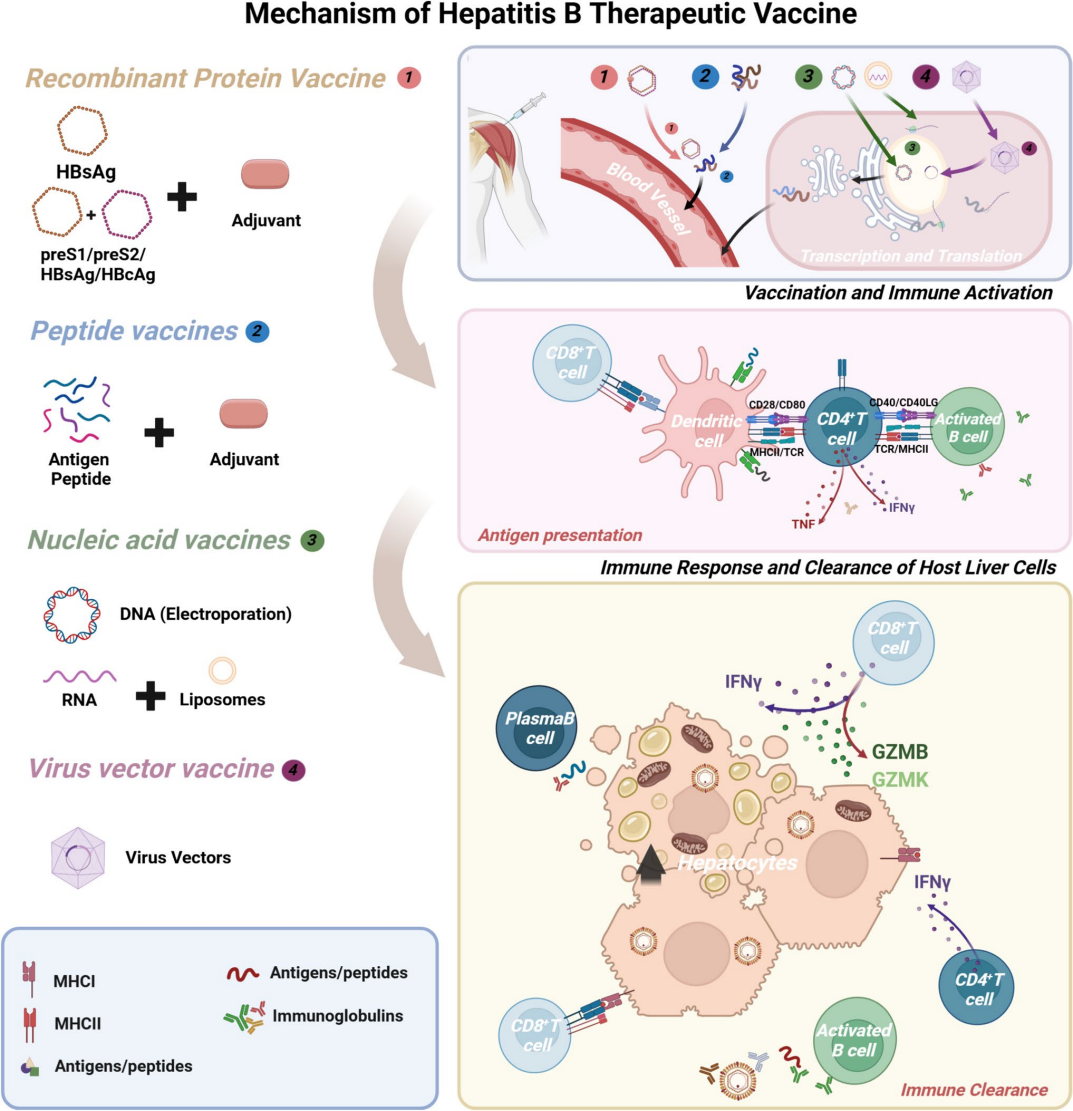
Schematic diagram of therapeutic vaccine mechanism

Currently, there are several therapeutic hepatitis B vaccines in clinical trial phase II or III (Table 2), including BRII-179, NASVAC, TVAX-008, GS-4774, VTP-300 and so on [3–8]. Therapeutic HBV vaccines can be categorized into recombinant protein, peptide vaccines, nucleic acid vaccines, and adenovirus vectors, all of which can achieve effective serum decline of HBsAg and HBV DNA. This article would discuss the different mechanisms, clinical efficacy, and future development directions of therapeutic HBV vaccines, offering a forward looking perspective for the functional treatment of HBV (Fig. 1).

**Table 2:** List of clinical stage therapeutic hepatitis B vaccines

No.	Drug type	Name	Effective components	Clinic trail	Critic inclusion criteria	Exclusion criteria co-infection	Primary Objectives	Participants	Phase	Country	Year(start)
1	Recombinant protein	NASVAC [4,5]	HBsAg, HBcAg	NCT01374308	HBsAg^+^ ≥ 6 months, HBeAg^-^&DNA ≥ 10^3^, HBeAg ^+^ &DNA ≥ 10^4^,	HCV, HDV, HIV, Liver cirrhosis, liver cancer, AID	Virological and or biochemical response	160	III	Cuba	2011
2		BRII-179 (VBI-2601) [3]	PreS1, PreS1, HBsAg	NCT06491563	HBV infection ≥ 6 months, BRII-179-experienced with anti-HBs ≥ 10 IU/mL, 100 < HBsAg ≤ 3000 IU/mL	HCV, HDV, HIV, liver cirrhosis, liver cancer	HBsAg seroclearance,levels of HBsAb	150	IIa/IIb(with PEG-IFNα)	America	2024
3		GS-4774 [7]	HBsAg, HBcAg, HBx	NCT01943799	HBsAg^+^ ≥ 6 months, HBV DNA- ≥ 1 year	HCV, HDV, HIV, Cirrhosis	Levels of HBsAg, HBsAb, HBeAg, HBeAb	178	II (with TDF)	America	2013
4		TVAX-008 [6]	HBsAg, HBcAg, CpG	CTR20202547	HBsAg^+^ ≥ 6 months,	HCV, HDV, HIV, TP, other liver diseases	Levels of HBsAg, HBsAb, HBeAg, HBeAb HBV DNA	47	II	China	2020
5	Lipopeptide vaccines	εPA-44 [30]	HBV epitope map	CTR20210513	HBsAg^+^ ≥ 6 months& DNA ≥ 2.0 × 10^4^ IU/mL, HBeAg ^−-^&Anti-HBs^-^, ALT at 2 and 10 times of the normal value	HCV, HDV, HIV, Liver cirrhosis, liver cancer, AID	HBeAg/ anti-HBe serological conversion	480	III(with Entecavir)	China	2021
6		ISA104 (HEB-PEP) [31]	Synthetic Long Peptide	NCT05841095	CHB with NUCs and HBV DNA < limit of quantification	liver cirrhosis	Levels of HBsAg, HBsAb, HBeAg, HBV DNA	24	I/II	French	2023
7	Nucleic acid vaccines	HB-110 [39]	preS1,HBsAg, HBcAg, HBx, Pol	NCT01641536	CHB with Entecavir ≥ 6 months; HBV DNA ≤ 300 copies/mL, ALT ≤ 2 times ULN	Presence of any other primary or secondary hepatic disease other than hepatitis B	AEs	9	I/II	America	2011
8	Adenovirus vector vaccine	VTP-300 [8]	PreC, HBcAg, Pol, preS1, preS2, HBsAg	NCT05343481	HBsAg^+^ ≥ 6 months with > 10 and ≤ 4,000 IU/mL, HBeAg^+^ and HBeAg^-^, HBV DNA ≤ 1,000 IU/mL	HCV, HDV, HIV, Liver cirrhosis, liver cancer, AID	Levels of HBsAg with a greater than 1 log HBsAg reduction	120	IIb	England	2022
9		HB400	HBsAg, HBcAg, HBeAg	NCT05770895	HBsAg ≤ 5000 IU/mL with antiviral ≥ 6 months	Not detailed	AEs	83	I	America	2023

## CHB immunological mechanism

The immunopathogenesis of CHB remains incompletely understood. In the early stage of HBV infection, it is related to the innate and adaptive immunity of the body. Firstly, the risk of chronicity is age dependent: approximately 90% of neonates and infants develop CHB, compared to 30% in children aged 1-5 years, and < 5% in adults, reflecting immune system maturation [9−12]. Immunomodulatory agents such as pegylated interferon-alpha (Peg-IFN-α-2a/2b) exert antiviral and immunoregulatory effects by inducing chemokines like CCL4, recruiting cytotoxic CD8⁺ T cells [13, 14]. However, antiviral therapies are unable to eliminate the cccDNA, restricting sustained virological control.

Compared to acute HBV infection, patients with CHB exhibit significantly reduced expression of effector molecules (e.g., Gzmb, Cxcr3, Ccr2, and Ccr5) in intrahepatic CXCR6⁺ CD8⁺ T cells, along with elevated expression of co-inhibitory receptors such as PD-1, TIGIT, TIM-3, and LAG3, indicating CD8⁺ T cell exhaustion [15]. Moreover, enhanced activity of the transcription factor CREM (cAMP-responsive element modulator) has been identified as a key mechanism contributing to the functional impairment of CD8⁺ T cells [15]. CD8⁺ T exhaustion undermines the HBVspecific cellular immune response and facilitates viral persistence, thereby sustaining chronic infection.

In addition, B lymphocyte mediated humoral immune responses are essential for the clearance of HBeAg and HBsAg. Anti-HBs antibodies play a critical role in preventing reinfection and maintaining long-term immune control. In CHB, B cells predominantly exhibit an atypical memory B cell (atMBC) phenotype, which is associated with impaired capacity to produce anti-HBs antibodies [16, 17]. Among the 5 patients who received bone marrow from anti-HBs^+^ donors, 2 achieved sustained HBsAg clearance, whereas none of the 16 patients who received bone marrow from HBsAg^+^ or seronegative donors cleared HBsAg (P < 0.05). This indicates that anti-HBs positivity can lead to sustained HBsAg clearance in patients with chronic HBV infection [18]. Moreover, the ability of B cells to internalize, process, and present HBcAg is significantly reduced in CHB, thereby compromising T cell activation and response. Numerous studies have shown that the use of B cell depleting agents, such as Rituximab, markedly increases the risk of HBV reactivation. Approximately 20% to 80% of HBsAg^+^ patients treated with these agents develop viral hepatitis, with a significantly elevated incidence of hepatocellular carcinoma [19, 20]^.^

Therefore, achieving functional cure fundamentally relies on the reconstitution of the immune system, particularly the restoration of HBV specific T and B cell functions. HBeAg and HBsAg seroconversion serve as key indicators of disease progression and therapeutic response in the natural history and clinical management of CHB.

## Recombinant protein vaccines

### HBsAg monovalent antigen vaccines

Recombinant protein vaccines achieve efficient expression of key antigenic proteins and induce specific immune responses with high safety. Recombinant protein therapeutic HBV vaccines are designed around conserved epitopes of the HBV HBsAg and HBcAg, attempting to activate or enhance specific immunity against HBV and break the immune tolerance in CHB.

In a phase I/II clinical trial conducted by GlaxoSmithKline plc (GSK), the therapeutic efficacy of 100 μg HBsAg with the AS02B adjuvant plus lamivudine was evaluated in patients HBsAg^ +^  ≥ 12 months, HBeAg^+^  ≥ 6 months and no interferon or antiviral therapy within 6 months. At the end of treatment (EOT) of 52w, there were no significant difference in HBeAg seroconversion and the decline of HBV DNA among all groups [21]. The BRII-179 (VBI-2601) is formulated using virus-like particles (VLPs) composed of Pre-S1, Pre-S2, and S proteins. The Phase II clinical trials (ACTRN12619001210167,NCT04749368) was conducted in CHB received NAs treatment for at least 6 months and HBsAg levels between 50 and 5000 IU/ml. The results showed that BRII-179 combination with PEG-IFN-α could induce T cell responses in most patients that secreted IFN-γ specific to S, Pre-S1, and Pre-S2 antigens, and 30% anti-HBs response, no significant HBsAg decline among all groupes [3]. At the 2025 APASL Congress held in Beijing, updated data from the ENSURE study phase II (NCT06491563) showed that 18 of 28 patients who received BRII-179 previously and achieved an anti-HBs response ≥ 10 IU/mL, with HBsAg level > 100 and ≤ 3000 IU/mL,  and then were subsequently treated with elebsiran (siRNA) plus Peg-IFNα. At the EOT 48w, the BRII-179 experienced group achieved 61% (11/18) HBsAg seroclearance vs 10% (1/10) in the control group. And 91% (10/11) subjects achieved HBsAg seroclearance among the anti-HBs responders maintained anti-HBs titers ≥ 100 IU/L [22]. The results show that BRII-179 experienced responders can enhance HBsAg loss. These findings underscore the advantage of therapeutic vaccine induced HBV specific immunity and highlighting the potential therapeutic vaccine as an essential immunomodulatory agent within functional cure regimens.

### HBsAg-HBcAg combined vaccines

In addition to HBsAg as an immune antigen, HBcA and HBeAg were confirmed to induce T cell independent/dependent antibody responses. HBcAg also modulates the specific immune responses to HBsAg and HBcAg in HBV transgenic mice, activating endogenous DCs without causing liver damage [23]. And the HBcAg/HBeAg specific Th2 cells in HBV transgenic mouse models could escape tolerance mechanisms and become activated [24]. Therefore, the combination of HBsAg and HBcAg antigen is believed to activate the specific immune response much better and offer new options for clinical cure and combined therapy.

The vaccine NASVAC in Phase III is the most promising product which is composed of 100 μg HBsAg and 100 μg HBcAg VLPs antigens and could rapidly stimulate mucosal immune mechanisms by nasal route. An open-label, randomized, controlled phase III clinical trial (NCT01374308) with the baseline HBsAg^+^ and HBeAg^-^/HBV DNA ≥ 10^3^ copies/mL, HBeAg^+^ /HBV DNA ≥ 10^4^ copies/mL, the results showed that the proportion of patients with HBV DNA < 250 copies/mL was similar between the NASVAC and Peg-IFN groups (59.0% vs 62.5%, p > 0.05) at EOT. At 24 weeks post-treatment, 57.7% patients in the vaccine group maintained HBV DNA < 250 copies/mL, compared with only 35.0% in the Peg-IFN group (p < 0.01), indicating that NASVAC confers a more durable virological suppression after therapy termination [4, 5]. Five years after EOT, follow-up of 60 patients showed that their HBV DNA levels decreased over time, with 8/12 HBeAg^+^ patients showing HBeAg seroconversion, and 55/60 patients had reduced serum HBV DNA levels, with 45/60 becoming HBV DNA negative in their serum and 40 patients had normalized ALT levels, and no patients developed HCC or cirrhosis [5, 25]. Long-term follow-up (5 years) showed no adverse effects on the liver, kidneys, nasal mucosa, etc. The results suggest that NASVAC immunotherapy is significantly safe and effective in suppressing HBV DNA and ALT, and in preventing the development of cirrhosis and HCC. While it needs to pay much attention on the HBsAg seroconversion mechanism. Combined treatment with antiviral drugs might be tried to explore the best functional cure with limited course of treatment.

Another therapeutic vaccine TVAX-008 is composed of HBsAg and HBcAg with CpG adjuvant (TLR9 receptor agonist). In HBV transgenic mouse models(HBV Tg), TVAX-008 effectively induces HBsAg/HBcAg specific humoral responses reducing serum HBsAg levels without causing liver damage and promotes HBsAgspecific Th1/Th2 immune responses and HBcAg specific Th1 immune responses with secreting IFN-γ [6]. So it is expected to break the CHB immune tolerance mechanism. In May 2023, Phase II clinical trials (CTR20202547) were initiated in patients with HBsAg^+^  > 6 months, which has been completed and the data are yet to be disclosed.

The Hepatitis B X protein (HBx) is essential for HBV to establish a robust infection and viral gene expression in host cells [26]. And HBx has been proved that it is the first to initiate the transcription and expression processes which are regulated by host nucleosomes [26]. The GS-4774 vaccine primarily expresses HBsAg, HBcAg and HBx [7]. In a phase II trial (NCT01943799) conducted in patients with HBeAg^+^ , mean HBsAg level at 2.5-3.1 log_10_ IU/mL after oral antiviral (OAV) for ≥1 year. After 6 doses, 5/151 patients achieved HBeAg seroconversion, and 3/50 patients in the 40YU treatment group had a ≥ 0.5 log_10_ IU/ml HBsAg decrease at 48w. GS-4774 could induce more significant HBcAg and HBX specific T cell response (*p* < 0.05), while it failed to reduce HBsAg level among OAV + GS-4774 groups from baseline to week 24 or 48 [27]. The results of phase I/II were not satisfactory and did not achieve exciting efficacy. And then, a global, multicentre, open-label phase II clinical trial (NCT02174276) involving 34 sites evaluated the efficacy and safety of combination therapy GS-4774 combined with Tenofovir dioxopyamate fumarate (TDF) in patients who were positive HBsAg > 6 months, HBV DNA ≥ 2000 IU/mL and had not received antiviral treatment within 3 months [28]. TDF with GS-4774 (at doses of 2, 10, or 40 YU) was administered subcutaneously every 4 weeks for a total of 6 doses as well as the phase II trial (NCT01943799). The results showed that CHB patient could been activated specific T cells, and the effect on CD8^+^ T cell activity was greater than that on CD4^+^ T cell activity, but not reduce HBsAg levels effectively [28]. To summarize, GS-4774 significantly increased cytokine production by HBV-specific CD8^+^ T cells but failed to significantly impact viral load or HBsAg levels and had limited effects on CD4^+^ T cells.

The above results indicate that recombinant protein vaccine including HBsAg and HBcAg can effectively activate specific immune responses. Although they cannot effectively reduce or clear the serum HBsAg levels, patients received vaccines have better prognosis outcomes compared to those treated with antiviral drugs or interferon only [4, 5, 25]. While some vaccines reported adverse events (AE) in clinical trials, such as GS-4774 is 92% (40 YU), 94% (10 YU), and 82% (2 YU), significantly higher than the 37% in the OAV-only group, most of these AEs were mild or moderate and did not impact treatment [27]. Overall, no serious AEs related to the vaccines were reported in the majority of cases, demonstrating good safety and tolerability of the recombinant protein vaccine.

## Peptide-based vaccines

Polypeptide vaccines are designed to target specific antigenic epitopes, thus exhibiting high specificity and safety. HepTcell developed by Altimmune is composed of nine synthetic peptides targeting HBV antigen epitopes, combined with IC31®, a TLR9 agonist. In a Phase I trail 60 HBeAg^-^ subjects received entecavir or tenofovir tolerated HepTcell well, and safety after three doses and successfully activated specific HBV cellular immune responses [29]. While the results of the Phase II (NCT04684914) clinical trial failed to meet the bar for further development, which conducted among IHC patients with qHBsAg levels ranging from 10 to 100 IU/mL and HBeAg^−^ within 12 months. Altimmune decided in June 2024 to cease the further research on HepTcell. The primary reasons for the failure may be insufficient efficacy to decline HBsAg.

εPA-44 based on the HBV antigen epitope map achieved positive results in Phase II study (NCT00869778) which the patients were HBsAg^+^  ≥ 6 months, HBV DNA ≥ 100,000 copies/ml, HBeAg^+^ and HBsAb^−^. The results showed 38.8% HBeAg seroconversion in 900 μg vs placebo (20.2%), 18.1% ALT normalization and HBV DNA < 2000 IU/mL in 900 µg, 14.3% 600 µg vs placebo 5.0% at the week 76. And in the 68 week extension stage, none (0/20) of the patients treated with 900 µg εPA-44 experienced serological recurrence [30]. εPA-44 was able to significantly increase the HBeAg seroconversion rate and decrease the HBV DNA level and a phase III trial is ongoing (ChiCTR2100043708) [30]. Although HBeAg seroconversion is considered an important therapeutic goal in CHB treatment, the lack of sufficient data on HBsAg seroconversion makes it unclear to guide functional cure. It needs to further explore the long-term effects of HBsAg seroconversion.

The another peptide vaccine ISA104 developed by ISA Pharmaceuticals utilizes synthetic long-polypeptide technology (SLP®) [31]. This project was in phase I (NCT05841095) in the CHB patients with HBV DNA < limit of quantification and may efficiently induce CD4^+^ and CD8^+^ T cell responses [31]. Peptide vaccine presents good safety and tolerability with mild side effects, such as injection site reaction and fatigue. Compared with natural pathogens or recombinant protein vaccines, peptide vaccines lack antigenic spatial epitopes. Moreover, HBV has high genetic heterogeneity, with various genotypes and subtypes, making it difficult to manufacture a peptide vaccine that provides broad protective effects. There is a need to explore more diverse and effective adjuvants or delivery systems to enhance the immunogenicity.

## Nucleic acid vaccines

Nucleic acid vaccines are primarily divided into DNA vaccines and RNA vaccines. As of now, only veterinary DNA vaccines have been approved for commercial production. With the breakthroughs in the thermal stability technology of mRNA vaccines have provided new impetus for vaccine research and development.

### RNA vaccines

Based on artificial intelligence algorithm, a therapeutic HBV mRNA vaccine delivered by LNP (lipid nanoparticle) primarily encodes the HBsAg protein [32, 33]. In HBV-AAV mice, the serum HBsAg level rapidly decreased after the 1st dose and achieved HBsAg seroconversion with high levels of anti-HBs after 3 doses. When challenged with the pAAV-HBV1.2 plasmid 60 days later, the anti-HBs levels increased and HBsAg was undetectable in the serum, demonstrating that the vaccine has a durable antiviral effect. Additionally, the levels of HBV cccDNA, total DNA, total RNA, 3.5 kb RNA, and serum HBV DNA, as well as the level of HBcAg in liver tissue, were significantly reduced [32]. These results show that the HBV mRNA vaccine can effectively and durably eliminate HBV and achieve long-term HBsAg seroconversion, providing broad prospects for the CHB functional cure and the prevention of HBV recurrence. Meanwhile, it needs to explore the safety and mechanism for eliminating HBV cccDNA. And mRNA vaccine encoding at phase I (NCT07051187) trail is conducted to evaluate the safety and effectiveness [34].

### DNA vaccines

The clinical results about DNA vaccines, for example, the pCMV-S2.S DNA plasmid developed by the Pasteur Institute and the dual plasmid HBV DNA developed by Guangzhou Baiyunshan Baidi Biomedical Co., Ltd., showed that the HBeAg seroconversion is generally low, and there is no significant difference in serum HBsAg levels among different groups, indicating that the therapeutic effect is generally modest.

The pCMV-S2.S DNA vaccine encodes the small (S) and medium (pre-S2 + S) proteins of the HBV envelop and was conducted on 70 CHB patients with undetectable HBV DNA in Phase I/II clinical trials(NCT00536627) [35]. The results showed that after discontinuation of NAs treatment, 97% patients in both groups experienced viral reactivation within a median of 28 days, while failed to restore anti-HBV immune response [35]. The dual plasmid HBV DNA vaccine utilizes electroporation as the drug delivery technology which could enhance the immune efficiency. The vaccine includes two plasmids, pcDNAS2.S and pcDNAIIF, which express HBV preS2, S antigens, as well as vaccine adjuvants human interleukin-2 (hIL-2) and human interferon-gamma (hIFN-γ), respectively [36]. The dual plasmid HBV DNA vaccine was conducted sequential combination therapy with lamivudine (LAM) in patients with HBsAg and HBeAg positivity for at least 6 months with HBV DNA > 10^6^ copies/mL (1.79 × 10^5^ IU/mL). The results of phase IIb trial (NCT01487876) showed that more patients in the vaccine group had HBV DNA > 2 log_10_IU/mL at week 12. Among the patients HBV DNA < 1000 copies/mL at week 12, the HBeAg seroconversion rate was (6/11) 54.5% in the vaccine group at week 36 after EOT (6/11) 54.5% vs (3/19, 15.8%, P = 0.042) in the control group [37]. Among the 111 recipients, 14 (12.6%) observed limited mild adverse events, while among the 118 patients who received the placebo, 15 (12.7%) observed limited mild adverse events, and there were no AEs which  lead to any withdrawal during the study period [37]. While the dual plasmid HBV DNA vaccine combined with entecavir, there was no statistically significant difference in HBsAg decline of all groups in a phase IIc (CTR20150841, CTR20150842) study [38]. The clinical outcomes of both vaccines are not satisfactory, the main reason may be that immune responses induced by HBV DNA vaccines are often transient and weak, making it difficult to maintain long-term antiviral effects.

And the HB-110 vaccine modified from HB-100 and was composed of mixed three plasmids, pGX10-S/L, pGX10-C/P, and pGX10-hIL-12 m, in a ratio of 2:1:1. The phase II trial (NCT01641536) was conducted on the patients with HBsAg^+^ and HBV DNA ≤ 300 copies/mL. The results of the HB-110 vaccine demonstrated that electroporation technology can enhance the efficacy and is four times that of the electric pulse method [39]. The combination of HB-110 and adefovir dipivoxil (ADV) could induce HBV specific T cell responses and achieve HBeAg seroconversion in CHB patients, but was weaker than that of HB-100, which was contrary to the results of animal model experiments [40]. Therefore, the subsequent therapeutic outcomes for HBV are not optimistic. These results indicate that DNA vaccines could stimulate and produce immune responses with good safety and tolerability. However, the immunogenicity of DNA vaccines is relatively weak, and they failed to achieve the expected experimental results, which has restricted their further development.

## Viral vector vaccines

Therapeutic hepatitis B virus vector vaccines constructed based on non-pathogenic viruses, attenuated live vaccine viruses, or non-pathogenic bacteria can utilize specific antigen genes to express corresponding proteins, thereby activating immune responses. The VTP-300 vaccine developed by the Vaccitech uses Chimpanzee adenovirus Oxford 1 (ChAdOx1-HBV) and Ankara vaccinia (MVA-HBV) as viral vectors. ChAdOx1-HBV contains almost the entire sequence of HBV proteins, including pre-core protein (PreC), core protein, polymerase with 8 point mutations, preS1, preS2, and S proteins [8]. The Phase I clinical trial (NCT04297917) was conducted on 10 healthy volunteers and 11 CHB with HBsAg^+^  < 4000 IU/m, HBV DNA < 40 IU/mL for ≥ 6 month. The results show that ChAdOx1-HBV has good safety in healthy volunteers and CHB, and due to the exhaustion of T cells in CHB, HBV-specific T cell response in CHB after vaccination is lower than that in healthy individuals [41]. It indicates that VTP-300 faces the issue of potential insufficiency in immunogenicity in humans, and the efficacy of subsequent studies may be affected by the baseline HBsAg levels of patients.

HB-400 developed by Gilead Sciences in collaboration with Hookipa Pharma utilizes the Lymphocytic Choriomeningitis Virus (LCMV) and the Pichinde Virus (PICV) as arenavirus backbones to express three highly conserved antigens of HBV. This alternating dual vector, non-replicating viral vector vaccine is designed to optimize and concentrate the immune response against the targeted antigens [42]. Activated antigen presenting cells (APCs) induce antigen specific CD8^+^ T cell immune responses through the MHC I pathway, under the influence of co-stimulatory factors such as CD80/CD86, and trigger the release of pro-inflammatory cytokines [42, 43]. A Phase I clinical trial (NCT05770895) is currently underway to primarily assess the safety and tolerability of repeated doses of HB-400 in healthy individuals and CHB patients who have HBsAg levels ≤ 5000 IU/mL.

LV-LHBs based on a Lentiviral vector (LV) and encodes the core, preS1, or large HBsAg (LHBs). The research shown promising results in HBV mouse models which indicated the effectiveness of LV-LHBs in inducing antigen specific T cells, and reducing serum HBsAg levels and HBV DNA load [44]. After a 34 weeks observation period, 60% (6/10) HBV persistent mice vaccinated achieved HBsAg seroconversion, and significant reduction in HBV-positive cells in the liver [44]. Additionally, two inactive HBsAg carriers experienced a continuous decline of HBsAg by −0.31 log_10_ IU/ml and −0.46 log_10_ IU/ml, and one patient showed a significant increase of peripheral L-HBs specific T cells [44]. The study suggests that the LV vector vaccine for HBV is feasible and potentially therapeutic. However, the clinical sample size in this experiment was small and given the genetic variability of HBV, it is necessary to analyze the therapeutic effects on pathogens from different genetic backgrounds.

## Discussion

The immunological mechanisms of CHB have not yet been fully elucidated. Currently, there is no effective therapeutic vaccine on the market, but they have shown great potential for immune regulation in long-term control of viral relapse. In the combination treatment of BRII-179 (VBI-2601) + Elebsiran (siRNA) + PEG-IFN-α, the BRII-179 received group achieved 61% (11/18) HBsAg clearance vs 10% (1/10) in the control group at the EOT 48w [22]. It indicates that the HBV specific immune response activated by therapeutic vaccines can improve HBsAg seroconversion. The long-term comparative study of NASVAC and Peg-IFN-α showed that at EOT 5 years, 55/60 (91.7%) vaccine group had reduced serum HBV DNA levels, with 45 cases below the detection limit, and 8/12 HBeAg^ +^ patients achieved HBeAg seroconversion [5, 25]. Compared with healthy individuals, the activation of T cell immune responses by VTP-300 is weaker in CHB [41]. Therefore, regulating immune responses to restore effective HBV specific T cell responses is a promising strategy for maintaining long-term antiviral efficacy in CHB.

Currently, immunotherapy is being applied in the treatment of HBV related liver cancer. The HBsAg specific T cell receptor (TCR) therapy (code: SCG101, NCT06617000) has shown rapid reduction in HBsAg levels within 3 days post infusion and clearance of HBsAg^+^ liver cells within 73 days, significantly lowering HBsAg levels and reducing tumor lesions by 70% [49]. Similarly, the CAR-T drug C-CAR031 (CTR20243113, NCT05155189), targeting GPC3, has demonstrated good safety and significant antitumor activity in liver cancer patients, with 14/24 patients achieved objective response and 91.3% disease controlled [50]. Additionally, Ori-CAR-001, in a clinical study of 9 patients with relapsed/refractory liver cancer, resulted in 4 cases of partial response and a disease control rate of 77.78%, with significant tumor shrinkage or even disappearance [51].While immunotherapy has achieved encouraging results for HCC, bringing hope to patients with advanced disease, challenges remain. Patients who have recovered from HBV are prone to HBV reactivation after reinfusion, which can induce hepatitis or liver injury. Furthermore, high affinity GPC3 specificity may exacerbate extra tumoral AEs and have serious or potentially fatal side effects. Therefore, the development of new targets on liver cancer cell surfaces and more extensive genetically engineered immune cells (such as CAR-NK, CAR-NKT, CAR-γδT, CAR-M, CAR-DC) is essential to provide more options for future personalized treatments.

T cell exhaustion induced by high levels of HBsAg results in immune tolerance [48]. Antiviral therapies have high relapse rates (20%) post-treatment, challenging sustained viral control [2]. HBeAg seroconversion usually indicates lower viral replication, HBsAg seroconversion, however, is a stronger marker of functional cure. Understanding the mechanisms of seroconversion is key to optimizing treatments and improving clinical cure rates in CHB patients. Currently, IFN treatment is the most promising regimen to achieve clinical cure. In IHC patients (HBsAg < 1500 IU/mL), the use of interferon therapy can achieve over 40% HBsAg clearance [46, 47]. Therapeutic vaccines could break immune tolerance and achieve long-term antiviral effects by activating HBV specific T cell and antibody responses. And now, many therapeutic vaccines use combination treatment with interferon (Supplementary Table S1) to enhance the body's immune response for increasing the rate of functional cure in clinical trials.

Recently, it was proved that HBx protein could activate viral gene transcription and replication under the action of host nucleosomes, promoting the persistent infection of HBV [26]. It provides a new target for the development of targeted treatment strategies. The mRNA vaccine encoding HBx, WGC-0201, initiated its Phase I (NCT07051187) clinical trial in July 2025 to evaluate the safety and effectiveness. Preclinical studies have demonstrated its good safety profile, ability to effectively clear HBV DNA, reduce HBsAg levels in serum and liver tissue, and enhance the proliferation of specific T lymphocytes [34]. Currently, the clearance of cccDNA and integrated HBV DNA is the key goal for complete cure of HBV infection, but no effective drugs have been approved yet. However, with the continuous maturation of gene editing technology, such as PBGENE-HBV and CRISPR/Cas9, it has brought new hope for clinical cure. These technologies, which precisely target and edit the viral genome, hold the potential to fundamentally eliminate the virus and provide a new path for the complete cure of HBV infection.

## Conclusion and prospect

“Functional cure” within a finite treatment duration has become the primary clinical goal for CHB treatment. Therapeutic vaccines activate HBV specific T cell and antibody responses for sustaining long-term antiviral effects, and the results of therapeutic vaccines combination with interferons studies show promise for achieving functional cure, enhancing specific immune functions and improving seroclearance efficiency in patients with low viral loads. Thus, clinical studies should conduct more detailed stratification of patients based on HBsAg, HBeAg levels, HBV DNA viral load, and age to facilitate targeted treatment guidance.

In the future, the integrated application of multiple novel technologies, methods, and strategies will emerge as a crucial direction for achieving the functional cure of HBV. Building on antiviral therapies, restoring or augmenting the patient's immune regulatory mechanisms, breaking immune tolerance, and ultimately eradicating cccDNA in the liver may offer genuine hope for a cure.

## Supplementary Information

Below is the link to the electronic supplementary material.

Supplementary file1 (DOCX 29 KB)
