# Supplementary material for: Do the therapeutic vaccines hold hope for the treatment of hepatitis B?
Source: Hepatol Int. 2025 Sep 27;19(6):1320–30. doi: 10.1007/s12072-025-10907-2 (PMC12714817; doi:10.1007/s12072-025-10907-2)
Supplement: Supplementary file 1 — Supplementary file1 (DOCX 29 KB) [file 12072_2025_10907_MOESM1_ESM.docx]

Supplementary Table1 Research on clinical treatment regimens combined with interferon

| **No.** | **Name** | **Combined with** | **Clinic trail ID** | **Phase** | **Critic inclusion Criteria** | **Patients** | **Country** | **Year** |
| --- | --- | --- | --- | --- | --- | --- | --- | --- |
| 1 | BRII-179+BRII-835（VIR-2218）+Peg-IFNα | Therapeutic vaccine + siRNA +interferon | CTR20243850 | Phase II | HBsAg^+^≥6months, HBVDNA^-^≥6months | 250 | China | 2024 |
| 2 | VIR-2218+Peg IFNα | siRNA +Interferon | NCT05970289 | Phase II (Active, not recruiting) | HBsAg^+^≥6months, NRTI therapy ≥6months | 75 | China | 2023 |
| 3 | VIR-2218+VIR-3434+PEG IFNα | siRNA+mAb±Interferon | NCT04856085 | Phase II (Active, not recruiting) | HBsAg^+^≥6months, NRTI therapy ≥2months | 244 | China | 2021 |
| 4 | AB-729+VTP300+NA±nivolumab( AB-729+PEG IFNα±NA) | siRNA+ Therapeutic vaccine ±PD-1 inhibitor | ACTRN12622000317796 | Phase IIa (Active, not recruiting) | HBsAg ≥100 IU/mL and < 5,000 IU/mL， HBeAg^+/-^, HBV DNA < 20 IU/mLwith NA suppressed | 40 | America | 2022 |
| 5 | JNJ-73763989+NA+Peg IFNα | siRNA+NA+Interferon | NCT05005507 | Phase II (Terminated) | HBsAg>5 IU/mL, HBeAg^-^ with anti-HBe^+^, HBV DNA＜60 IU/mL at least 6 months | 1 | America | 2021 |
| 7 | EYP001a+NA+Peg IFNα | FXR激动剂+NA+Interferon | NCT04365933 | Phase IIa (Completed) | HBeAg ^+^/HBV DNA ≥ 20,000 IU/mL, HBeAg^-^/HBV DNA ≥ 2,000IU/mL, HBsAg ≥ 2.5 log10 IU/mL | 20 | French | 2020 |
| 8 | ASC42+NA+Peg IFNα | FXR激动剂+NA+Interferon | NCT05107778 | Phase II (Completed) | HBsAg^+^≥6months, HBV-DNA- | 43 | China | 2022 |
| 9 | REP 2139+Peg IFNα+TDF | HBSAg抑制剂+Interferon+NA | NCT02726789 | Phase II (Completed) | HBsAg^+^, anti-HBs- | 5 | Canada | 2012 |
| 11 | REP 2165+Peg IFNα+TDF^1^ | HBSAg抑制剂+Interferon+NA | NCT02565719 | Phase II (Completed) | HBsAg> 1000 IU / ml, HBV DNA > 10000 copies / ml, HBeAg-, anti-HBe^+^ | 40 | Canada | 2016 |
| 12 | GSK3228836+Peg IFNα+NA | AOS+Interferon+NA | NCT04676724 | Phase II (Completed) | HBsAg^+^≥6months and >100 IU/mL, HBV DNA <90 IU/mL | 108 | England | 2021 |
| 13 | Hepalatide+Peg lFNα（Hepalatide+Peg lFNα+TAF) | Entry inhibitor +Interferon | NCT05244057 | Phase II (Completed) | HBsAg +/ HBV DNA+≥6 months,HBeAg-, HBV DNA< LLQD | 8 | China | 2022 |
| 14 | RO7049389+NA±Peg IFNα | Capsid inhibitor+NA+Interferon | NCT02952924 | Phase I (Completed) | HBsAg+≥6months, HBeAg +/HBV DNA ≥ 20,000 IU/mL, HBeAg-/HBV DNA ≥ 2,000IU/mL, | 192 | America | 2016 |
